# Replicating the ‘seductive allure of neuroscience explanations’ effect in a classroom experiment and an online study

**DOI:** 10.1098/rsos.241120

**Published:** 2024-12-11

**Authors:** Pearl Amber Väth, Jakob von Petersdorff, Christof Neumann, Roger Mundry, Julia Fischer

**Affiliations:** ^1^ Cognitive Ethology Laboratory, German Primate Center–Leibniz Institute for Primate Research, Göttingen, Germany; ^2^ Department of Primate Cognition, Georg-August-University Göttingen, Göttingen, Germany; ^3^ Leibniz ScienceCampus Primate Cognition, German Primate Center–Leibniz Institute for Primate Research, Göttingen, Germany

**Keywords:** classroom experiment, neuroscience, replication, seductive allure, science communication

## Abstract

The ‘seductive allure of neuroscience explanations’ effect refers to the observation that superfluous neuroscience information (SNI) added to an explanation can bias judgements of information quality. We report the results of a classroom experiment to sensitize undergraduate students to that issue. In contrast to previous studies, students rated good explanations without SNI the highest. Inspired by these observations, we set out to conceptually replicate the original study using an online experiment that allowed us to directly assess the statistical interactions between explanation quality, the presence of SNI and expertise levels. In this preregistered study, participants (*n *= 430) with varying levels of expertise rated the quality of good and bad explanations, with or without SNI. Irrespective of the presence of SNI, participants across all expertise levels rated good explanations more favourably than bad ones. Still, the differences were surprisingly small, and the variation in rating was high. We also found a statistically significant interaction between the impact of SNI and expertise, with SNI boosting ratings mostly in participants with less expertise (*p *< 0.001), corroborating previous findings. Developing a curriculum that trains students to distinguish between actual explanations and ‘crap’ would ultimately also sensitize teachers and experts that produce and review scientific information.

## 1. Introduction

Neuroscientific information carries a unique persuasive appeal. Even when not directly relevant, it can present an argument with an air of authority and scientific credibility [1–3]. As neuroscience continues to gain prominence in public discourse, understanding its influence on beliefs, attitudes and behaviour becomes increasingly important [4]. The ‘seductive allure of neuroscience explanations’ (SANE) effect highlights how neuroscience explanations and brain imagery can influence information quality and persuasiveness judgements. In a seminal study on the SANE effect, Weisberg and colleagues explored the impact of adding irrelevant neuroscience information to explanations in three experiments [3]. The authors first presented participants with different psychological phenomena. They then offered different explanations and asked participants to rate their satisfaction. The explanations varied with regard to quality and the presence of superfluous neuroscience information (SNI) that did not add to the understanding of the phenomenon. Good explanations provided a reason (mechanism) for a given psychological phenomenon, while bad explanations merely repeated the results, in other words. These good and bad explanations were presented with or without superfluous neuroscience information in a 2 × 2 design.

The three experiments by Weisberg and colleagues involved different groups of participants: study 1 was conducted with novices (*n* = 81), study 2 with students in a cognitive neuroscience class (*n* = 22) and study 3 with neuroscience experts (*n* = 48). Studies 1 and 3 treated the quality of explanation as a within-subject variable and the presence of neuroscience explanation as a between-subject variable. Study 2 treated the quality of the explanation and the presence of neuroscience information as within-subject variables due to the small sample size [3]. Across all participant groups, good explanations received higher ratings than bad ones, and superfluous neuroscience information boosted the rating of bad explanations in novices and students but not in experts [3]. In contrast, experts rated good explanations with SNI as less satisfying than those without.

Several studies have shown that it is not mere explanation length (associated with adding SNI) that affects the positive rating of explanations combined with SNI [5–7]. A follow-up study showed that participants rated explanations containing reductive information more favourably across several scientific disciplines [1]. These authors also showed that participants with higher scientific literacy or those who had taken science courses demonstrated higher proficiency in distinguishing between good and bad explanations. In a large meta-analysis, Bennett and McLaughlin confirmed the presence of the SANE effect across 60 experiments from 28 publications with a sample size of 13 800 participants. Considering only the ratings of laypeople, they found a mild though highly significant impact of neuroscience information on evaluating information [8].

Here, we report the outcome of two studies. Study 1 comprises a classroom experiment that we had developed to alert students to ‘crap’ [9] and how to distinguish valid explanations from mere repetitions of results. The classroom experiment was built on the study by Weisberg *et al*. [3], and one item (see table 1) of their stimulus material was used. Over the years, we observed substantial variations in the aggregated ratings of the students and, overall, a pattern that deviated from the one described in the original study. We thus decided to embark on a conceptual replication study of the original investigation. A conceptual replication refers to testing the same hypothesis using a different approach. Conceptual replications are informative concerning the generalizability of results [10]. The aim of study 2 was to extend systematic investigations of the SANE effect into the German-speaking community and to place our findings from the classroom experiment into a broader context. We hypothesized that people can generally distinguish good explanations from bad ones and that increasing expertise diminishes the seductive allure of neuroscience information. Thus, we predicted that irrespective of the presence of SNI, participants would rate good explanations higher than bad ones and that this effect increases with higher expertise. We further predicted that the statistical effect of SNI diminishes with higher expertise, resulting in a three-way interaction between explanation quality, SNI and expertise.

**Table 1.** Item 1 of 18, as established by Weisberg *et al*. [3] and then slightly edited, with good and bad explanations in the absence and presence of superfluous neuroscience information.

Phenomenon: Researchers created a list of facts that about 50% of people knew. Subjects in this experiment read the list of facts and had to say which ones they knew. They then had to judge what percentage of other people would know those facts. Researchers found that the subjects responded differently about other people’s knowledge of a fact when the subjects themselves knew that fact. If the subjects did know a fact, they said that an inaccurately large percentage of others would know it too. For example, if a subject already knew that Hartford was the capital of Connecticut, that subject might say that 80% of people would know this, even though the correct answer is 50%. The researchers call this finding ‘the curse of knowledge’.		
	explanation	
	good	bad
without SNI	The ‘curse of knowledge’ happens because subjects have trouble switching their point of view to consider what someone else might know. People mistakenly project their own knowledge onto others.	The ‘curse of knowledge’ happens because subjects make more mistakes when they have to judge the knowledge of others. People are much better at judging what they themselves know.
with SNI	**Brain scans indicate that this ‘curse’ happens because of the frontal lobe brain circuitry, known to be involved in self-knowledge**. Subjects have trouble switching their point of view to consider what someone else might know, mistakenly projecting their own knowledge onto others.	**Brain scans indicate that this ‘curse’ happens because of the frontal lobe brain circuitry, known to be involved in self-knowledge**. Subjects make more mistakes when they have to judge the knowledge of others. People are much better at judging what they themselves know.

The neuroscience information is highlighted here, but subjects did not see such markings. The full table with all items used in study 2 can be found at https://osf.io/4mwje/.

## 2. Methods

### 2.1. Classroom experiment (study 1)

The classroom experiment was part of the lecture ‘good scientific practice’ by the last author. This lecture is mandatory for second- or third-year bachelor students of biology and biochemistry at the University of Göttingen. The experiment was first run in 2008 and every year since then. Within a session on science communication, we discussed the need for clear and accessible communication and the importance of considering one’s audience when communicating. The cover story was that students would now learn about the ‘curse of knowledge’, the observation that one’s knowledge may affect the attribution of knowledge to others and how this effect may affect one’s communication.

Students were presented with the text (translated into German; see electronic supplementary material) of item 1 of the phenomena used by Weisberg and colleagues in their original study (table 1). Mirroring the 2 × 2 design of the original study, four different types of explanations (good and bad explanations with or without SNI) were printed separately on paper slips, mixed and distributed among the students. Students were then asked to rate their satisfaction with the explanation (without consulting each other) on a scale from −2 to 2 (simplifying the original design that used a rating from −3 to 3 to facilitate data collection in the classroom). Each student rated only one explanation. We then collected the ratings, typed the numbers into a prepared spreadsheet, and visualized the results in front of the students. Subsequently, we debriefed the students and compared their ratings to those in the original study. During the COVID-19 pandemic (2020 and 2021), data collection took place online, and students participated in multiple ratings; we did not use these data in the present analysis. The data presented here span 2008–2022 and involved ratings by *n* = 887 students. We collected no personal information (age and gender) from the students, and participation was voluntary. We did not fit a statistical model to these data but illustrated the raw data with the means and the bootstrapped confidence intervals.

### 2.2. Online experiment (study 2)

The study design conceptually followed the design of the original study [3], using a within-subject design throughout. Study 2 was preregistered (https://doi.org/10.17605/osf.io/7bz84). Each participant was presented with four out of the 18 scientific phenomena previously established [3]. After preregistration, we slightly edited the stimulus material. Specifically, we presented the neuroscience information in a separate sentence to keep the wording of the actual explanation constant in the conditions with and without SNI.

Moreover, we edited the content that referred to gender and race-related issues to avoid repeating gender stereotypes and issues with using the term ‘race’ in German, where it has a pejorative connotation. The original, adapted and translated versions of the survey items are deposited at https://osf.io/4mwje/. The study was hosted on the survey platform ‘shinyapps.io’ by Posit Software (https://www.shinyapps.io). Initially, the planned sample size was *n* = 1000, but due to time constraints, we had to terminate the study when we had reached 584 participants. We had not done power analyses to determine the sample size but had based the estimate loosely on the findings of the classroom experiment, where a sample size of *n* = 400 yielded a stable pattern. Given the addition of a further factor (expertise), we opted for a larger sample size that we deemed feasible. Ultimately, however, we were too optimistic regarding the recruitment of participants.

Participants accessed the survey through a shared link. This link was distributed across various platforms, including social media, email distributions and websites. Participants started the survey by selecting their preferred language (German or English) and then consented to using their data. Next, they received a concise overview of the study procedure, providing a brief description without revealing too much detail (electronic supplementary material).

Participants were asked to provide information on their expertise by choosing one of the six levels described in table 2. Each participant was then presented with four different phenomena (items), which previously had been randomly paired with one of the factor combinations of interest (good/bad and with or without SNI), such that each participant was confronted once with each of the four-factor combinations of interest. Participants were required to rate the quality of the explanation on a 7-point scale, ranging from −3 to +3. Between rating the items, participants were asked to enter their age in years and gender (options: female, male, non-binary and prefer not to say) and solve a simple maths question. The latter question was added to ensure that participants were still paying attention. This information was not used in the analysis. The survey took approximately 10 min to complete. In the end, participants could contact the study’s first author via email to receive updates on the study’s results.

**Table 2.** Level of expertise queried from the participants who gave at least one rating.

level	expertise	*N*
0	no high school diploma	37
1	high school diploma	124
2	general university degree	112
3	degree in natural sciences	73
4	PhD in neuroscience and related fields	33
5	postdoc, lecturer or professor in neuroscience and related fields	51

We randomized the order in which we presented the factor combinations and items to each participant. However, we restricted the randomization such that the sample was as close as possible to being counterbalanced concerning (i) the frequency with which items were presented, (ii) the frequency with which items were combined with each of the four combinations of explanation quality and the presence of SNI, (iii) the frequency with which items were presented at the first, second, third and fourth position to participants, and (iv) the frequency with which the four combinations of explanation quality and the presence of SNI were presented to the participants.

After excluding data entries with missing values (i.e. lacking any quality rating), the original sample size of 584 participants was reduced to 430. The final dataset comprised 1624 ratings from 430 participants, rating 18 phenomena. Three hundred and eighty-six participants completed all four questions, 11 participants rated only three of the four explanations, 14 rated only two, and 19 rated only one explanation. Unlike the preregistered plan, we also included participants with less than four ratings in our analysis. The reason for doing so was that we had reached much fewer than the originally planned 1000 participants and thus wanted to maximize the use of the ratings available. We also fitted the model for the subset of the participants who had completed all four ratings; the results showed no noticeable differences (see electronic supplementary material, tables S3–S5).

### 2.3. Data analysis

To estimate the extent to which satisfaction ratings were related to explanation quality, the presence of SNI and the level of expertise, we fitted a generalized linear mixed model [11]. It included fixed effects of explanation quality and SNI, level of expertise and all their interactions up to the third order. We included a fixed effect of first language (German or English) and of trial number to control for their possible effects on the ratings. Since the response was a rating, we used a cumulative logit link, also known as ordinal model [12].

#### 2.3.1. Equation 1: simplified model equation


$$\begin{array}{c} rating∼quality^{*}presence\;of\;SNI^{*}education\;level+language+trial.nr+\\ \left(1|item\;ID\right)+\left(1|participant\;ID\right) \end{array}$$

The full model equation can be found in the electronic supplementary material. The ‘education level’ in the models corresponds to the ‘level of expertise’ in the article.

Most participants rated up to four items, and as items were used multiple times (between 83 and 106 times), we included random intercept effects for these two factors. These control for possible variation among participants and the items concerning the average rating. We included all theoretically identifiable random slopes to keep the type 1 error rate at the nominal level of 0.05 and avoid an ‘overconfident’ model [13,14]. The random slopes were those of explanation quality and the presence of SNI within participant ID and those of explanation quality, SNI, first language, level of expertise, their interactions up to order three and trial number within item ID. Initially, we also included a random slope of trial number within participants and item and parameters for the correlations among random intercepts and slopes. However, to avoid an overly complex model and convergence issues, we removed them from the model again.

As an overall test of the statistical effects of explanation quality, SNI, level of expertise and their interactions and to avoid cryptic multiple testing [15], we compared the full model as described above with a null model lacking explanation quality, SNI, level of expertise and their interactions in the fixed effects part. For the full-null model comparison, we used a likelihood ratio test [16]. We tested the statistical significance of individual fixed effects by dropping them from the model, one at a time, and comparing the likelihood of the resulting reduced models with that of the full model (R function drop1). The full-null model comparison indicated a statistically significant effect of the predictor variables, but the three-way interaction between the presence of SNI, explanation quality and explanation quality was not statistically significant; so we removed the three-way interaction from the model. The removal allowed us to assess the contributions of the three two-way interactions between the presence of SNI, explanation quality and level of expertise. Two of these were not statistically significant (quality*SNI and quality*expertise), so we removed them, too. This removal allowed us to assess the statistical effect of the remaining interaction between SNI and expertise and the main effect of quality in the final model. Furthermore, the removal allowed us to obtain unconditional estimates of the effects [17], which otherwise cannot be obtained for the main effects involved in interactions or the two-way interactions involved in three-way interactions. We report the results of all models in the main text and the electronic supplementary material.

We fitted the model in R (v. 4.4.0 [18]) using the function clmm of the package ordinal (v. 2023.12-4 [19]). We included the level of expertise as a numeric predictor, although it is an ordered factor. We did so for three reasons: first, doing so reduced model complexity considerably, particularly as we would have had to add random slopes for that factor and the interactions with it within the item. Second, it turned out that the level of expertise only moderately varied with satisfaction ratings, in the sense that, per combination of the level of expertise, question quality and the presence of SNI, the actual satisfaction ratings varied a lot and generally covered the entire space of the available ratings. Finally, plotting the data and the model did not reveal hints that including the level of expertise as a quantitative predictor would have led to an apparent mismatch between the model and the data.

Before fitting the model, we *z*-transformed the level of expertise and trial number to a mean of zero and a standard deviation of one to ease model convergence. We manually dummy-coded and centred all factors before including them as random slopes. We determined model stability by dropping individual participants and individual items, one at a time, fitting the full model to each of the subsets, and finally comparing the range of estimates obtained with those we had gathered for the full dataset. This procedure revealed the model to be of good stability. We estimated the confidence limits of model estimates and fitted values using a parametric bootstrap (*n* = 1000 bootstraps). Collinearity among the predictors, assessed using variance inflation factors (VIF) [20] was not an issue (maximum VIF: 1.2; determined for a standard LMM lacking the interactions using the function vif of the package car v. 3.1-2 [21]).

In the figures presenting the results of this analysis, we included ‘fitted values’ with confidence limits. These were derived as follows: we first determined fitted values (also for each bootstrap) in terms of the probability of observing a given satisfaction rating for a given constellation of values of the predictors. We then determined the weighted mean of the response, treating it as a quantitative variable, with the weights being the probabilities of the individual ratings. Given the ordinal nature of the satisfaction ratings, we know that treating the response as a quantity is not fully appropriate. However, adding such ‘fitted values’ and their confidence limits gives an intuitive illustration of the model’s findings.

Of the 72 possible item–explanation combinations (18 items × 4 explanation types), each combination occurred 16 to 32 times in the survey. The ages of the participants ranged from 16 to 83 years. The genders in the sample consisted of *n* = 286 persons who identified themselves as females, *n* = 129 as males, *n* = 4 persons who identified themselves as non-binary and *n* = 9 who preferred not to provide information on their gender. Over half of the participants selected German (*n* = 246) as their language, while 184 participants chose English. The sample consisted of participants across all six levels of expertise (table 2). Data and code are deposited at https://osf.io/4mwje.

## 3. Results

### 3.1. Study 1

**Figure 1.**
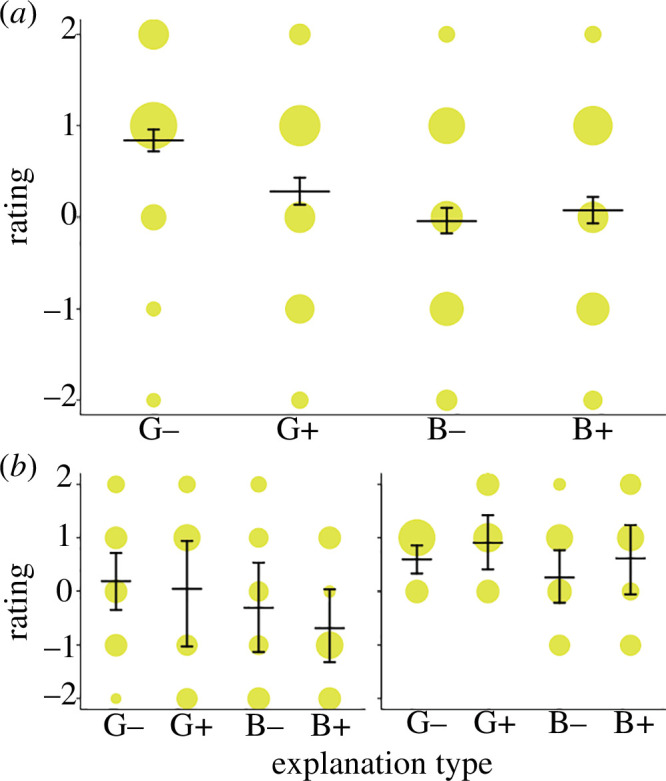
Satisfaction rating for good (‘G’) and bad (‘B’) quality explanations in the absence (‘−’) and presence of (‘+’) superfluous neuroscience information (SNI) in the classroom experiment. The area of the dots corresponds to the proportion of ratings per explanation type category. (*a*) Pooled satisfaction ratings across all study years (*n* = 887 participants). (*b*) Exemplary ratings for the years 2014 (*n* = 62) and 2019 (*n* = 48) to illustrate the variation between cohorts. Horizontal lines with error bars are fitted means with 95% confidence limits.

For the item used with undergraduate students in the classroom experiment, good explanations without SNI yielded the highest ratings and good explanations with SNI yielded the second-highest ratings. The compound rating was 0.83 for good explanations without SNI, 0.28 for good explanations with SNI, −0.04 for bad explanations without SNI and 0.07 for bad explanations with SNI (figure 1*a*). However, there was considerable variation between the student cohorts (figure 1*b*).

### 3.2. Study 2

In the online survey, we found a clear statistical association between explanation quality, presence of SNI, level of expertise and one or several interactions with participants’ rating behaviour (full-null model comparison, likelihood ratio test: LRT, χ^2^ = 66.3, d.f. = 7, *p* < 0.001). Electronic supplementary material, table S1, provides the full model output. As the predicted three-way interaction between explanation quality, SNI and level of expertise was not statistically significant, we removed it to assess the association between the remaining two-way interactions and the rating. Of these, the interaction between SNI and level of expertise yielded a statistically significant association (χ^2^ = 17.0, d.f. = 1, *p* < 0.001). The other two-way interactions between explanation quality and SNI, as well as explanation quality and level of expertise, were not statistically significant (electronic supplementary material, table S2). We, therefore, removed these non-significant two-way interactions to assess the statistical main effect of explanation quality.

**Figure 2.**
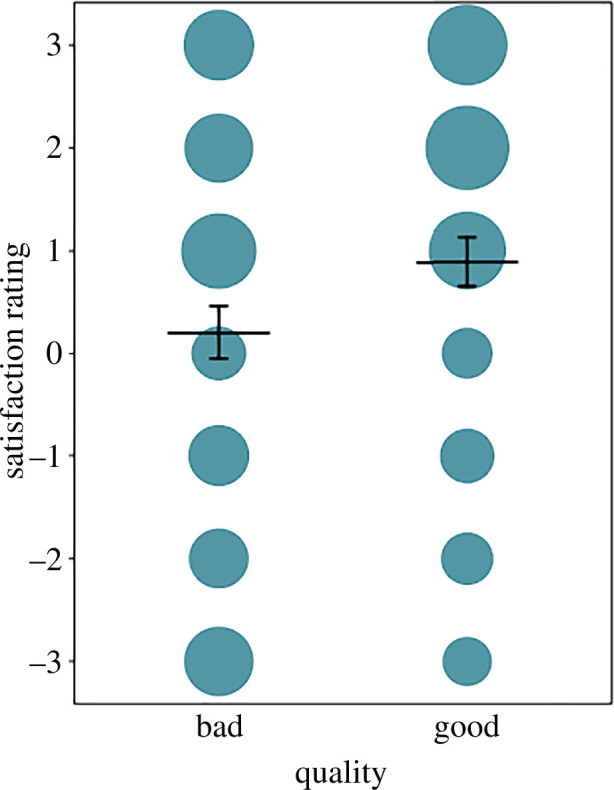
Satisfaction rating as a function of explanation quality. The area of the dots corresponds to the number of data points with identical values in satisfaction rating and explanation quality (range = 63 to 190, *n* = 1624 ratings). Horizontal lines with error bars represent fitted average ratings and 95% confidence limits (obtained from the reduced model lacking all non-significant interactions).

**Figure 3.**
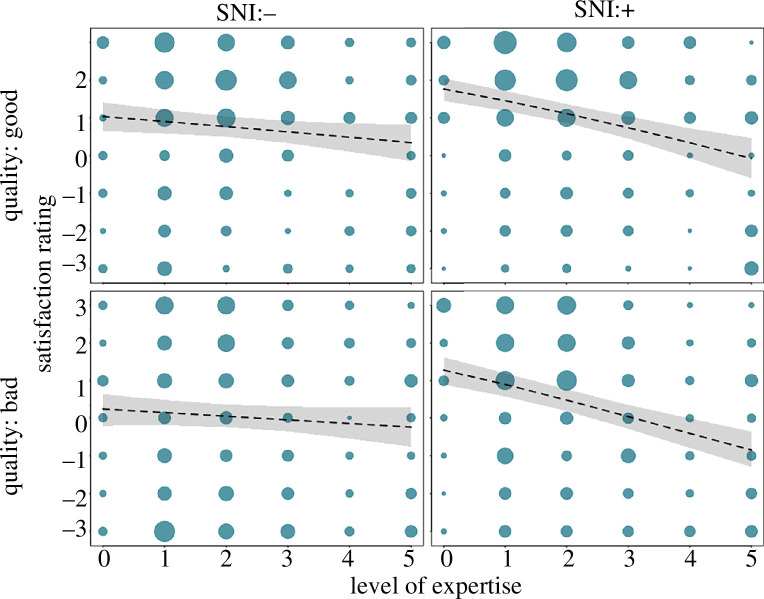
Satisfaction rating for good (upper) and bad (lower) quality explanations as a function of the level of expertise in the absence (left) and presence (right) of superfluous neuroscience information (SNI). The areas of the dots correspond to the number of data points for level of expertise, satisfaction level and SNI presence within each of the two explanation qualities (range = 1 to 35, *n* = 1624 ratings). The dashed lines with grey polygons represent the fitted averaged rating and their 95% confidence limits (obtained from the full model).

The final model revealed that good explanations were rated higher than bad ones (effect of quality: χ^2^ = 14.6, d.f. = 1, *p* < 0.001; see table 3 for the full model output; figure 2). Furthermore, at lower levels of expertise, the presence of SNI was associated with higher ratings than when SNI was absent, and the statistical effects of the presence of SNI decreased with increasing levels of expertise (figure 3).

**Table 3.** Results for fixed effects part of the reduced model lacking all non-significant interactions.

term	estimate	s.e.	CLlower	CLupper	χ^2^	d.f.	*p*
−3|−2	−2.174	0.207	−2.528	−1.851	—	—	—
−2|−1	−1.272	0.196	−1.589	−0.956	—	—	—
−1|0	−0.579	0.192	−0.886	−0.267	—	—	—
0|1	−0.078	0.191	−0.388	0.225	—	—	—
1|2	0.932	0.193	0.635	1.244	—	—	—
2|3	2.164	0.204	1.850	2.512	—	—	—
quality	0.699	0.146	0.475	0.914	14.604	1	<0.001
SNI	0.326	0.093	0.149	0.504	—	—	—
level of expertise	−0.192	0.083	−0.357	−0.040	—	—	—
language	−0.291	0.150	−0.543	−0.032	3.630	1	0.057
trial no.	0.169	0.047	0.081	0.264	11.628	1	0.001
SNI: level of expertise	−0.446	0.095	−0.616	−0.253	17.014	1	<0.001

Indicated are fixed effects estimates, standard errors, 95% confidence limits, significance tests and the range of estimates obtained when dropping individual participants and items, one at a time. All factors were dummy-coded with the reference levels being ‘bad’ (quality), ‘present’ (SNI, ‘superfluous neuroscience information’) and ‘English’ (language). All covariates were *z*-transformed to a mean of 0 and a standard deviation of 1; the mean and standard deviation of the original variables were 2.223 and 1.454 (level of expertise) and 2.455 and 1.120 (trial number).

**Figure 4.**
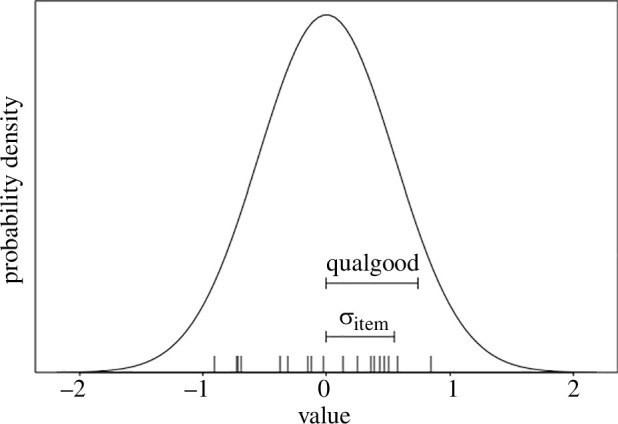
Illustration of the relative strength of the fixed main effect of explanation quality and the random intercept effect of the item. Vertical lines above the *x*-axis depict the estimated best linear unbiased predictors (BLUPs) for the random intercepts of the 18 different items. The depicted distribution is the probability density of BLUPs, which are assumed to be random samples from a normal distribution with a standard deviation as estimated for the random intercepts effect of the item (σ_item_, mean = 0, estimated s.d. = 0.55; the *x*-axis is depicted in the link space and the results shown are taken from the full model). In addition, we show the estimated fixed effect of quality (good versus bad, 0.68). Note that the variation in ratings due to different questions was of about the same magnitude as the difference in ratings of good and bad explanations.

There was considerable variation in ratings in relation to the items used in the study. Figure 4 shows the distribution of the best linear unbiased predictors (BLUPs) for the 18 items in the study. BLUPs ranged from −0.91 to 0.84. For comparison, the effect of explanation quality in the full model was 0.74. Interestingly, the pattern was very similar for item 1, which was used in the classroom and the online study. For this comparison, we extracted the ratings for level of expertise 2–4 (*n* = 209), which broadly corresponded to the levels expected of the students in the classroom experiment. Good explanations without SNI received the highest ratings (1.56 in the classroom experiment (maximum value: 2) and 0.89 in the online study (maximum value: 3)), good explanations with SNI received the second highest ratings (1.00 and 0.28), while bad explanations received similarly poor ratings (without SNI −0.33 and −0.04; with SNI −0.40 and 0.07, respectively).

## 4. Discussion

Both in the classroom experiment and in the online study, participants rated proper explanations higher than mere repetitions of the results. In the classroom experiment, which was based on one item only, a clear preference for a good explanation without SNI emerged, but only after a sample size of about *n* = 400 was reached. We attribute the variation between the cohorts mainly to stochastic effects, as we observed no systematic changes over the years. These results highlight the danger of jumping to false conclusions when inferences are based on a small sample.

In the online study, the addition of SNI was differentially linked to satisfaction ratings for persons with different levels of expertise: participants with less expertise rated bad explanations with SNI higher than those without SNI. Broadly, we corroborated the findings from previous studies on the SANE effect. There were, however, also some differences compared to the original study. While the original study [3] identified a significant statistical interaction between explanation quality and the presence of SNI (tested separately for novices, students and experts), we did not find evidence for such an interaction (electronic supplementary material, table S1). Moreover, in contrast to the original results, where experts rated bad explanations similarly irrespective of the presence or absence of SNI [3], experts in our study rated bad explanations with SNI worse than those without SNI. Otherwise, the pattern was generally similar (see electronic supplementary material, table S6, for details).

Our study revealed substantial differences in satisfaction ratings across the different items. Although Weisberg *et al.* [3] reported that subjects tended to respond similarly to all 18 items (Cronbach’s α = 0.79), the variation in ratings for different items in our study partly exceeded that of the differences between good and bad explanations. Note that we could not calculate Cronbach’s α for our dataset due to differences in the study design. Future studies need to consider such variation; it may also be necessary to check the stimulus material in greater depth before usage.

The minor discrepancies between our study and the original one may be due to several not mutually exclusive reasons, including stochastic effects, the inclusion of a second language (German), the slight editing of the items and explanations, differences in the design of the study (within- and between-subject designs) and the total number of ratings per participant. The absence of significant interactions between explanation quality and the presence of SNI and between explanation quality and the level of expertise may also be attributed to differences in statistical analyses. This study used a cumulative logit link model (CLLM), whereas the original study employed repeated measure ANOVA to assess statistical significance [3]. The advantage of CLLMs is that they do not assume normally distributed and homogeneous residuals, which are unlikely to be met with a bound and discrete response [22]. Moreover, they correctly limit fitted values and their confidence limits to the bound space of the response [22]. Yet, despite this range of possible sources of divergence, our findings are broadly comparable to those of previous studies, supporting the reliability and generalizability of the SANE effect [3,7,8]. Furthermore, none of the statistical results should be conceived as dichotomous, and it is always necessary to pay attention to the strength of the association.

The SANE effect has been attributed to neuroscience’s ability to offer reductive explanations [1–3,23]. Reductionist explanations provide insights into fundamental principles, rendering neuroscience explanations fitting for understanding psychological phenomena [5,24]. Considering the esteemed status of neuroscience as a scientific discipline and the tendency for scientific jargon to create a false sense of comprehension, it is reasonable to suggest that the allure of neuroscience may be driven by its prestige, aligning with the ‘prestige of science’ hypothesis [5,24]. There is ample evidence that expertise moderates the SANE effect [8]. Individuals who exhibited lower levels of reflection and reduced proficiency in verbal fluidity and numeracy were found to be more susceptible to accepting what could be termed ‘bullshit’ [25,26], leading individuals to mistake vagueness for profundity [27,28]. Such individuals were also likelier to judge pompous explanations as accurate and meaningful despite being hollow [27]. However, if individuals receive specific training on the SANE effect in their education, they may be more likely to detect it [1,3,7,27].

What we found striking (not only in our results) was the relatively small differences in the ratings of good and bad explanations. The mean ratings for the good explanations differed very little: they were 0.89 in our study and 0.88 in the original one; greater differences were observed in the ratings of the bad explanations, which were 0.20 in our study and −0.28 in the original one [3]. In other studies, good explanations received ratings of 0.70 [5] and 1.23 [1], higher than bad ones. Hopkins *et al*. [23] explored the link between expertise and rating behaviour in more detail. They found that expert participants rated good explanations (mean = 1.53, s.d. = 1.56) significantly higher than bad explanations (mean = −0.66, s.d. = 1.97), but there was considerable variation. Notably, the ‘bad’ explanations were not explanations at all but simple descriptions of the results, in other words. Yet, in our study, even some experts with several years in academia rated such ‘explanations’ highly with two or three points (figure 2).

Why were experts unable to distinguish between good and bad explanations more accurately than non-experts, even though expertise should improve the ability to evaluate explanations critically? In a previous study by Goldberg & Thompson-Schill [29], biology professors demonstrated less accuracy in their responses to statements about biology that contradicted basic principles than statements aligning with them. Known as the ‘curse of expertise’, experts tend to overestimate their knowledge in their specialized field [30]. Like the ‘illusion of explanatory depth’ in laypeople, expertise can create the illusion of competence within experts, leading individuals to believe they have a deeper understanding of a particular topic than they do [30–32]. Developing a curriculum that trains students to distinguish between actual explanations and ‘bullshit’ would ultimately also fire up the ‘crap detectors’ [9] of teachers and experts.

For science communicators, insights into the SANE effect and its variants underscore the responsibility of communicators to present information accurately and transparently without relying on jargon to influence perception [1,23,33,34]. As emphasized by Silas *et al.* [34], there is a pressing need for public health communication to prioritize clarity, simplicity and accessibility. They conducted a study that explored how explanations with scientifically irrelevant neuroscience affect intentions to vaccinate against COVID-19, demonstrating a significant impact of poor scientific communication on vaccination intentions [34].

Additionally, it was suggested that public health efforts are at risk of being sabotaged by misinformation that successfully uses technical language to persuade people to believe nonsensical explanations [34]. Contrary to the notion that technical language lends credibility, the data suggest that clear, simple and straightforward information is ultimately more effective in science communication than pompous language [33–35]. Moreover, we need to improve how we communicate the uncertainty of scientific results within and beyond the scientific community.

## Data Availability

The online study was preregistered [36] and all study materials, data and code are deposited at OSF [37]. Supplementary material is available online [38].
